# Supplementary material for: A novel emergency medical services protocol to improve treatment time for large vessel occlusion strokes
Source: PLoS One. 2022 Feb 25;17(2):e0264539. doi: 10.1371/journal.pone.0264539 (PMC8880856; doi:10.1371/journal.pone.0264539)
Supplement: Attachment — Submitted filename: Response to Reviewers Plos R2 12_13_21.docx [file pone.0264539.s002.docx]

Dear Editor,

Thank you for the reviewers’ comments. We are pleased to resubmit our article *A Novel Emergency Medical Services Protocol to Improve Treatment Time for Large Vessel Occlusion Strokes* with the responses listed below.

The authors made some efforts to improve the quality of the manuscript, but there is still lack of data and robust statistical analysis. As explained by the author “ Time from arrival via EMS to administration of tPA improved from median time of 59.8 minutes when the protocol was not in use to 43 minutes during the protocol (p<0.01).” I cannot see were this affirmation comes from. This data should be demonstrated through a table of case-control analysis. Because the analysis of modified transfer times was conducted during 5 months, I would suggest to compare this data with a hystorical control group of at least 5 months in the same period of the last year.

It is of outmost importance to incorporate number of patients treated with iv fibrinolysis as well as door-to-needle time (DNT) to iv fibrinolysis between both groups. “Table 1. Patient Characteristics” should be changed to a comparison analysis of historical control group vs analyzed group.

**We added a table 1 to show this for period from Oct-Feb 2019/2020 compared to Oct-Feb 2020/2021. When we used a historical control (as opposed to 5 months pre protocol and 5 months during protocol).**

The most important part of the manuscript is about reduction of times of treatment based on your protocol, in page 12 “(data with times and diagnoses included in S1) I guess that this means supplementary material S1 but I cannot find any table or figure in S1 including these KEY data.

**We included Table S1 at the end of the body of the manuscript (as opposed to a separate upload) to hopefully facilitate reviewer’s ability to see the data.**

So I ask again about my two previous questions to be answered
“Please include door-in-door-out (DIDO) times of each group, and reference the work from “Ambulance waiting and associated work flow improvement strategies of Eva Gaynor” We included DIDO times for the transferred patients add added the suggested reference.
WHERE IS THIS DATA?

**The reference to Eva Gaynor is in paragraph 3 of the discussion section, sentence 2.**

**Column 10 of Table 3 outlines the DIDO times for the stroke patients who were transferred. If the reviewer is referring to DIDO times for control patients, as discussed in the manuscript this data does not exist. The pilot study occurred in a time period when the EVT suite for the sending facility was being remodeled. In the year prior, there were no DIDO times because the patients were not being transferred. Similarly, DIDO times are not commonly collected for non-stroke emergent transfers as this is not a common metric in other pathologies.**

Did your protocol may help to improve times of intravenous fibrinolysis with or without LVO ? Can you provide data of door-to-needle time in those eligible for thrombolysis and can you compare these patients with a historical control group? We included further data on time to intravenous fibrinolysis during the protocol and before the protocol.
WHERE IS THIS DATA?

**As mentioned above, we added a table 1 to show this for period from Oct-Feb 2019/2020 compared to Oct-Feb 2020/2021. The time to intravenous fibrinolysis is additionally described in paragraph 1 of the results section.**

On the other hand the part of results that includes literally comments in pages 13 to 17 “ “ , I suggest to be included in a supplementary material because it does not improve the quality of the manuscript.

**We respectfully submit that a mixed methods approach is legitimate and highlights the important provider reactions to the pilot of a radically different protocol from that commonly used. For other groups attempting to pursue or operationalize a similar protocol in their system, understanding cultural context and reactions is important, not supplemental.**

The aim of the study should be to incorporate the protocol “Standby-for-Transfer” to improve times of treatment for iv fibrinolysis and transfer times of LVO strokes, so the results and the discussion should be related to this purpose.

**We did not find that the protocol improved times to fibrinolysis but we attempted to further emphasize the focus on improved time-to-EVT in the discussion section. We hope that by adding the new Table 1 in the results section we adequately addressed this concern regarding the results.**

Sincerely,

Nancy Glober, MD

Administration Offices: 720 Eskenazi Ave, FT 3^rd^ Fl. FOB, Indianapolis, IN 46202 • Phone: (317) 880-3900 • Fax: (317) 880-0545
Education Offices: 1701 N. Senate Ave, AG012, Indianapolis, IN 46202 • Phone: (317) 962-5975 • Fax: (317) 963-5394

Department of Emergency Medicine • Indiana University School of Medicine
